# A Gin4-Like Protein Kinase GIL1 Involvement in Hyphal Growth, Asexual Development, and Pathogenesis in *Fusarium graminearum*

**DOI:** 10.3390/ijms18020424

**Published:** 2017-02-16

**Authors:** Dan Yu, Shijie Zhang, Xiaoping Li, Jin-Rong Xu, Zachary Schultzhaus, Qiaojun Jin

**Affiliations:** 1 State Key Laboratory of Crop Stress Biology for Arid Areas, Northwest A&F University, Yangling 712100, China; yudan@nwsuaf.edu.cn (D.Y.); zsjw4501@163.com (S.Z.); wy20105366@163.com (X.L.); 2 Department of Botany and Plant Pathology, Purdue University, West Lafayette, IN 47907, USA; jinrong@purdue.edu; 3 Department of Plant Pathology and Microbiology, Texas A&M University, College Station, TX 77840, USA; schultzz@tamu.edu

**Keywords:** wheat scab, wheat head blight, *Gibberella zeae*, conidiogenesis, virulence, septation

## Abstract

*Fusarium graminearum* is the main causal agent of *Fusarium* head blight (FHB) on wheat and barley. In a previous study, a GIN4-like protein kinase gene, *GIL1*, was found to be important for plant infection and sexual reproduction. In this study we further characterized the functions of *GIL1* kinase in different developmental processes. The Δ*gil1* mutants were reduced in growth, conidiation, and virulence, and formed whitish and compact colonies. Although phialide formation was rarely observed in the mutants, deletion of *GIL1* resulted in increased hyphal branching and increased tolerance to cell wall and cell membrane stresses. The Δ*gil1* mutants produced straight, elongated conidia lacking of distinct foot cells and being delayed in germination. Compared with the wild type, some compartments in the vegetative hyphae of Δ*gil1* mutants had longer septal distances and increased number of nuclei, suggesting *GIL1* is related to cytokinesis and septation. Localization of the GIL1-GFP fusion proteins to the septum and hyphal branching and fusion sites further supported its roles in septation and branching. Overall, our results indicate that *GIL1* plays a role in vegetative growth and plant infection in *F. graminearum*, and is involved in septation and hyphal branching.

## 1. Introduction

*Fusarium graminearum* (teleomorph *Gibberella zeae*) is a major causal agent of *Fusarium* head blight (FHB) or scab of wheat and barley [1]. In addition to yield losses, FHB caused by this pathogen often reduces grain quality and results in mycotoxin contamination [2]. One of the mycotoxins produced by *F. graminearum* is deoxynivalenol (DON), which is a potent protein synthesis inhibitor in eukaryotic organisms [3]. DON is also toxic to plant cells. In fact, the *TRI5* trichodiene synthase gene that is essential for DON biosynthesis is the first virulence factor characterized by molecular studies in *F. graminearum* [4,5,6]. The *TRI5* deletion mutant is still pathogenic and causes typical FHB symptoms on inoculated wheat kernels but it fails to spread via the rachis to nearby wheat kernels on the same wheat heads.

In the past decade, molecular genetics and functional genomics studies have characterized over a hundred of genes that are important for plant infection in *F. graminearum*, including a number of genes encoding different transcription factors, protein kinases, lipases, and metabolic enzymes [7,8,9,10,11,12,13,14,15,16,17,18]. Whereas most of these genes, similar to *TRI5*, are not required for the initiation of infection, but important for disease spreading, and several of them are essential for the initiation of infection, such as *GPMK1* and *MAP1* [19,20]. However, many of these mutants blocked in the key signal transduction pathways, unlike the mutants defective in trichothecene production, have pleiotropic phenotypes, suggesting the co-regulation of infection processes with growth and cellular developments by well conserved signaling cascades [8,21]. In the systemic functional study of the *F. graminearum* kinome, a total of 42 protein kinase genes were found to be important for plant infection. Mutants deleted of these genes were significantly reduced in virulence or non-pathogenic [13]. Thirty-two of them also had over 30% reduction in growth rate. One of them is FGSG_08701 (reannotated to FGSG_16988 in MIPS database) that encodes a protein kinase homologous to the GIN4 kinase in *Saccharomyces cerevisiae* [22]. Deletion of FGSG_08701 resulted in reduced growth, conidiation, and virulence in *F. graminearum* [13]. The FGSG_08701 deletion mutant was also defective in sexual reproduction and had increased tolerance to oxidative stress [13].

In *S. cerevisiae*, GIN4, KCC4 and HSL1 are three closely-related protein kinases that have the kinase domain at the N-terminus and a long, less conserved C-terminal region [23,24]. They have overlapping functions in cell cycle but also retain their own specific functions. GIN4 functions in septin organization, mitosis, and probably in regulating microtubule stability [22,25]. Deletion of *GIN4* leads to a striking reorganization of the septins [22]. In contrast, the Δ*kcc4* and Δ*hsl1* single mutants and Δ*kcc4*Δ*hsl1* double mutants all display essentially normal phenotypes [24]. Loss of *GIN4* in cells that are dependent upon CLB2 causes the formation of highly elongated buds. The Δ*kcc4* cells showed a multi-budded cell shape at the stationary phase, and Δ*hsl1* mutants showed a mild elongated-bud phenotype in grown to high cell density, indicating that all three of these protein kinases are related to cell polarity [22,24,26]. The inhibitory activity of SWE1 on CDC28 is counteracted by the activity of HSL1, GIN4, and KCC4 proteins during the cell cycle of *S. cerevisiae*, but only HSL1 appears to play a direct role in SWE1 regulation [24,26].

In *Schizosaccharomyces pombe*, CDR1 and CDR2 are two protein kinases orthologous to GIN4, HSL1, and KCC4 of *S. cerevisiae* [27]. Similar to GIN4, CDR1, and CDR2 act as the mitotic inducers by negatively regulating the activation of the WEE1 kinase (an ortholog of SWE1). However, unlike CDR1 that acts directly on WEE1 to regulate mitosis, the role of CDR2 in cell cycle regulation is more complex [28,29]. Although, GIN4 regulates septin organization, CDR1 and CDR2 have not been linked to septin function [27]. The *Candida albicans* genome contains two genes homologous to *S. cerevisiae* GIN4 and HSL1 [30]. CaGIN4 is required for the formation of the septin ring, but not the basal septin band, and is also required for the transition from pseudohyphae to hyphae. CaHSL1 is not required for septin ring organization or septum formation although it regulates pseudohyphal formation [30]. In *Ashbya gossypii*, another Saccharomycetales yeast, morphological and behavioral differences in the septin rings require the ELM1 and GIN4 kinases [31].

Although GIN4 homologues are conserved in filamentous ascomycetes, only the GIN4 of *Aspergillus nidulans* has been characterized [32]. The *AnGIN4* mutant was reduced in asexual development but displayed an early onset of sexual reproduction. In this study we further characterized the functions of the *F. graminearum* FGSG_08701 gene (named *GIL1* for GIN4-like 1). Our results showed that *GIL1* is involved in hyphal growth, conidiogenesis, septation and plant infection in *F. graminearum*.

## 2. Results

### 2.1. GIL1 Is Important for Normal Hyphal Growth

The *F. graminearum* FGSG_08701 gene is predicted to encode a 1136-amino acid protein kinase that has the highest similarity to GIN4 but is also highly similar to KCC4 and HSL1 of *S. cerevisiae*. GIN4, KCC4, and HSL1 are three paralogous protein kinases in the budding yeast [26,33,34]. Therefore, in this study we named the FGSG_08701 protein as GIL1 (GIN4-Like 1). Whereas *S. pombe* has two [27], *F. graminearum* and other filamentous ascomycetes analyzed in this study all have only a single *GIN4*-like gene (Figure S1).

**Figure 1:**
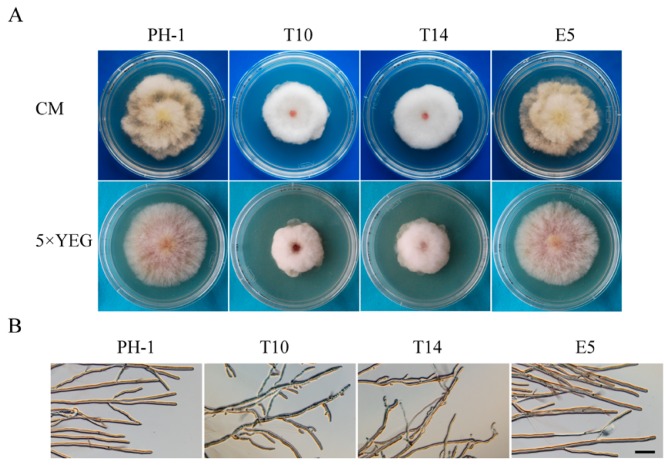
Growth defects of the Δ*gil1* mutants. (**A**) Colony of the wild-type (PH-1), Δ*gil1* mutants (T10 and T14), and ectopic strain (E5) were grown on complete medium (CM) and 5× YEG 5× YEG (Yeast Extract Glucose) agar plates. Photographs were taken after incubation for three days; and (**B**) edges of PH-1, T10, T14, and E5 colonies formed on 1/2 CM plates were examined for hyphal growth and branching. The Δ*gil1* mutants were increased in hyphal branching. Bar = 100 µm.

The *GIL1* gene replacement construct was generated by the split-marker approach and transformed into the wild-type strain PH-1 in a systemic characterization of the *F. graminearum* kinome study [13]. On potato dextrose agar (PDA) plates, the Δ*gil1* mutants displayed reduced growth and formed whitish, compact colonies with more fluffy aerial hyphae than the wild type strain PH-1. In this study, two independent gene replacement mutants, T10 and T14, were further confirmed by Southern blot analysis (Figure S2). When assayed for hyphal growth and colony morphology on complete medium (CM) and 5× YEG mediums, in comparison with PH-1, growth rate was reduced approximately 20% and 30% in the Δ*gil1* mutants, respectively (Table 1). Similar defects in colony morphology were observed in the mutants on CM and 5× YEG(Yeast Extract Glucose) as on PDA (Figure 1A; Table 1). Microscopic examination showed that the Δ*gil1* mutants were irregular in hyphal branching and produced curvy hyphae (Figure 1B). Thus, *GIL1* is important for vegetative growth and colony morphology in *F. graminearum*. The Δ*gil1* mutants appeared to be increased in hyphal branching and hyper-branching may be related to the formation of compact colonies from the mutants.

### 2.2. The *Δ*gil1 Mutants Produce Straight, Longer Conidia Lacking Typical Foot Cells

**Figure 2:**
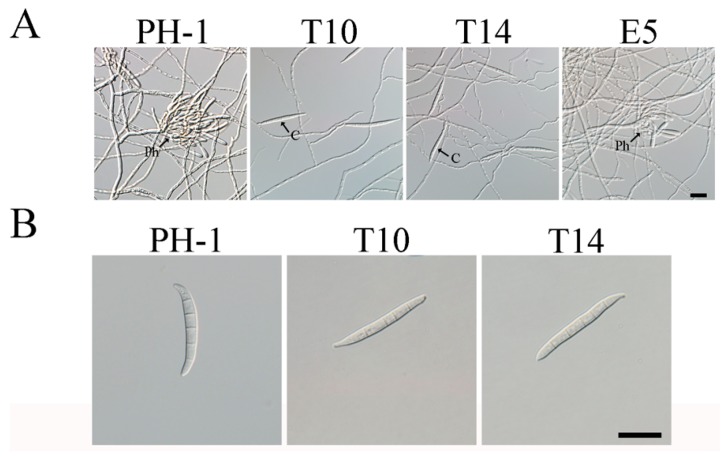
Conidiogenesis and conidium morphology defects of the Δ*gil1* mutants. (**A**) Five-day-old CMC cultures of the wild type (PH-1), Δ*gil1* mutants (T10 and T14), and ectopic strain (E5) were examined for conidiogenous structures. C, conidium; Ph, phialide. Bar = 10 µm; and (**B**) typical conidia of PH-1 and Δ*gil1* mutants. Bar = 20 µm.

Similar to the previous study [13], the Δ*gil1* mutants were reduced approximately 38% in conidiation compared to the wild type (Table 1). To elucidate the possible causes for the reduction of condiation, we assayed phialide formation of the mutants in five-day-old CMC (carboxymethylcellulose) cultures. Microscopic examination showed that the Δ*gil1* mutants often formed conidia directly on short hyphal branches or hyphal tips instead of phialides, suggesting the involvement of *GIL1* in phialide development (Figure 2A). In addition, we noticed that most of the Δ*gil1* conidia had abnormal morphology. Instead of forming typical *Fusarium* macroconidia, the Δ*gil1* mutants produced straight conidia that lacked typical foot cells (Figure 2B) and tended to be longer than the wild-type conidia (Table 1).

### 2.3. The *Δ*gil1 Mutants Are Delayed in Conidium Germination but Increased in Germ Tube Branching

**Figure 3:**
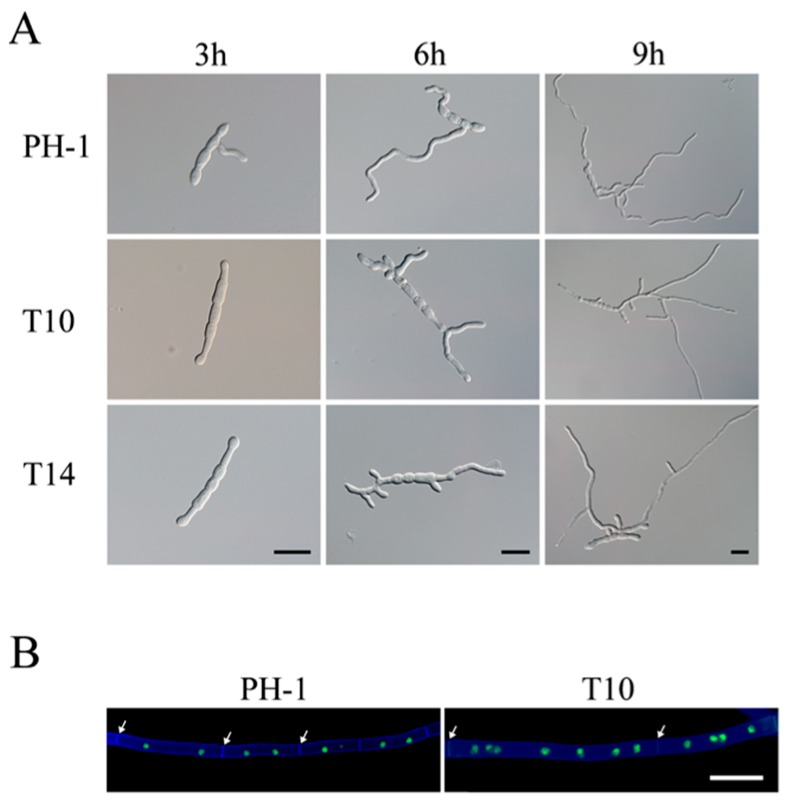
Defects in conidium germination and cytokinesisin the Δ*gil1* mutants. (**A**) Conidia of the wild type (PH-1) and Δ*gil1* mutants (T10 and T14) were incubated for 3, 6, and 9 h in YEPD medium. Bar = 20 µm; and (**B**) Vegetative hyphae from transformants of PH-1 and the Δ*gil1* mutant T10 expressing the H1-GFP construct were stained with Calcofluor white (CFW) and examined by the epifluorescence microscopy. Septa are marked with arrows. Bar = 10 µm.

Due to their morphological defects, conidia of the Δ*gil1* mutants were assayed for possible defects in germination. After incubation at 25 °C for 3 h in liquid YEPD (Yeast Extract Peptone Dextrose) medium, conidia of the wild-type had germinated and produced germ tubes longer than the width of conidia (Figure 3A). Under the same conditions, over 95% of the mutant conidia had barely visible or very short germ tubes (Figure 3A). At 6 h, short germ tubes formed by the mutants began to branch, but hyphal branching was not observed in the wild type (Figure 3A). After incubation for 9 h or longer, germ tubes of the Δ*gil1* mutants produced more branches or branching sites than those of PH-1 (Figure 3A). Therefore, the Δ*gil1* mutants were increased in germ tube branching, consistent with the hyper-branching phenotype observed at colony edges (Figure 1B).

### 2.4. GIL1 Is Involved in Cytokinesis in Vegetative Hyphae

To assay for possible defects in septation and nuclear distribution, we transformed the H1-GFP fusion construct [35] into the Δ*gil1* mutant T10 and the wild type strain PH-1. Vegetative hyphae of the transformants of PH-1 or T10 expressing H1-GFP were stained with Calcofluor White (CFW) as described [13]. When observed with an epifluorescence microscope, the vegetative hyphae of Δ*gil1* mutant were wider, and approximately 25% compartments have longer septal distances than that of the wild type. Moreover, the compartments of the mutant hyphae contained increased number of nuclei than that of the wild type (Figure 3B). These results indicate that deletion of *GIL1* likely resulted in cytokinesis and septation defects in *F. graminearum*.

### 2.5. The *Δ*gil1 Mutants Are Defective in Responses to Various Environmental Stresses

**Figure 4:**
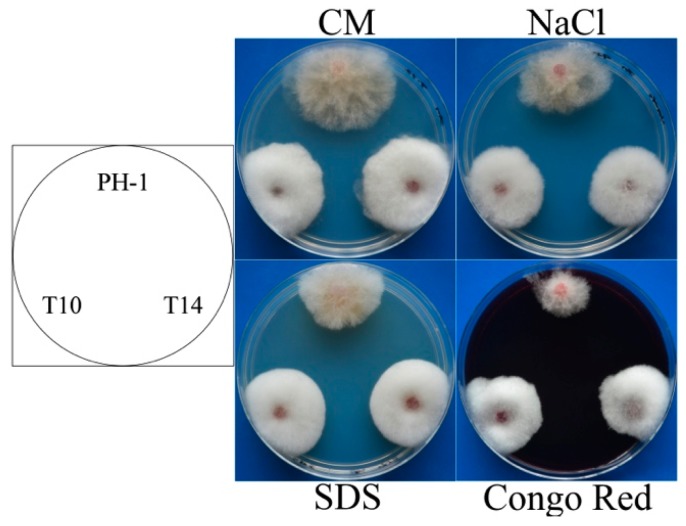
Assays for vegetative growth of the Δ*gil1* mutants in the presence of different stresses. Cultures of the wild-type (PH-1) and the Δ*gil1* mutants (T10 and T14) grown on regular CM, CM with 0.7 M NaCl, 0.01% SDS, or 300 µg/mL Congo Red. Photographs were taken after incubation at 25 °C for three days.

The *GIL1* deletion mutants had increased tolerance to oxidative stress in *F. graminearum* [13]. In this study, we further assayed colonial growth of the Δ*gil1* mutants on CM plates with three different chemicals that cause hyperosmotic, cell wall, and membrane stresses. In comparison with the wild-type, Δ*gil1* mutant stains had similar reduction in growth rate on CM plates with 0.7 M NaCl (Figure 4), indicating that GIL1 is likely dispensable for responses to hyperosmotic stress. However, the Δ*gil1* mutants grew faster than the wild type in the presence of 0.01% SDS or 300 µg/mL Congo Red (Figure 4). Therefore, deletion of GIL1 may also increase tolerance to cell membrane and cell wall stresses in *F. graminearum*.

### 2.6. GIL1 Plays a Critical Role in Plant Infection in F. graminearum

**Figure 5:**
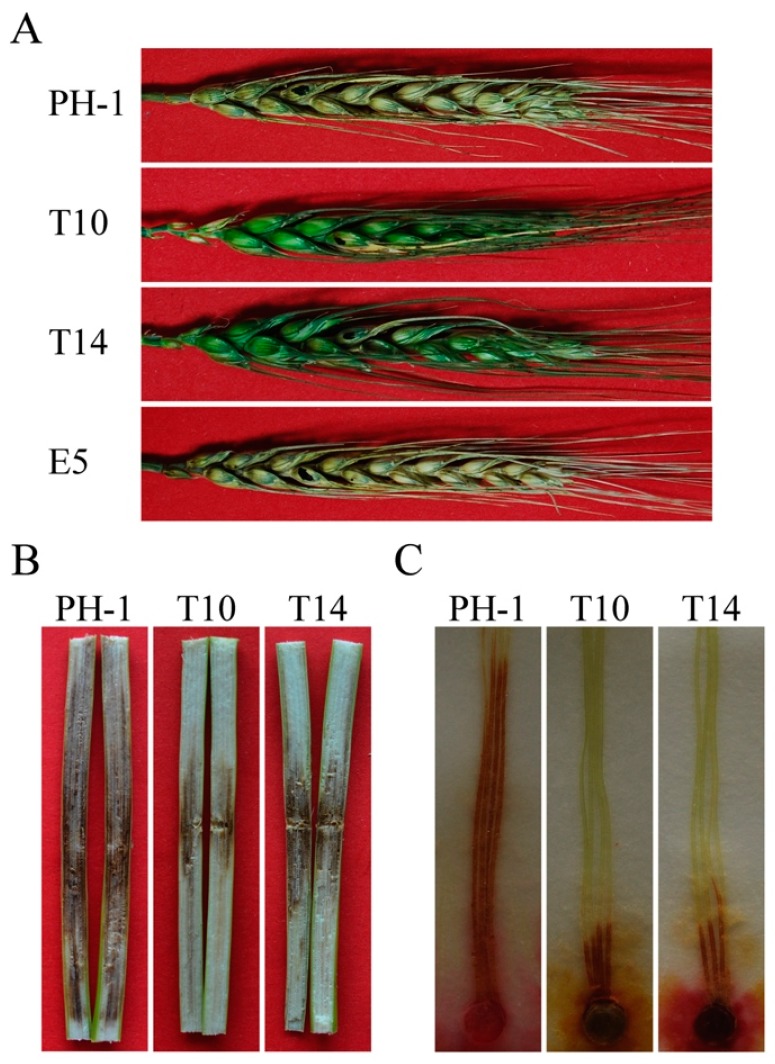
Infection assays with wheat heads, corn stalks, and corn silks. (**A**) Flowering wheat heads inoculated with the wild type (PH-1), the Δ*gil1* mutants (T10 and T14), and an ectopic strain (E5); (**B**) corn stalks inoculated with PH-1 and the Δ*gil1* mutants; and (**C**) corn silks inoculated with PH-1 and the Δ*gil1* mutants.

The Δ*gil1* mutants showed more servere defects in virulence on wheat heads in the current study than reported in the kinome study [13]. In infection assays with flowering wheat heads of susceptible wheat cultivar Norm, only the spikelets drop-inoculated with the Δ*gil1* mutants developed scab symptoms 14 days post-inoculation (dpi). The mutants failed to spread to nearby spikelets (Figure 5A). Under the same condition, the wild type caused typical head blight symptoms in the inoculated kernels and spread to other spikelets on the same heads, indicating that the Δ*gil1* mutants were significantly reduced in virulence, likely due to the defects in spreading from diseased kernels through the rachis to nearby spikelets. Because of the striking difference from the previous study, we repeated the infection assays on corn stalks and silks. In infection assays with corn stalks, the wild type caused extensive stalk rot in the pith of inoculated plants 14 dpi. On plants inoculated with the Δ*gil1* mutants, stalk rot symptoms were restricted to only a small area near the inoculation sites (Figure 5B). In infection assays with corn silks, the Δ*gil1* mutants also caused only limited discoloration near the inoculation sites (Figure 5C). These results confirmed that *GIL1* is important for plant infection in *F. graminearum*.

### 2.7. Localization of *GIL1-GFP* Fusion Proteins to Branching Point and Septa

To determine its localization, we first generated the *GIL1*-GFP fusion construct under the control of its native promoter and transformed it into the wild type PH-1. Unfortunately, the resulting transformants that were confirmed by PCR analysis to contain the *GIL1*-GFP construct had no GFP signals in conidia, germ tubes, hyphae, and ascospores, indicating that the expression level of *GIL1*-GFP may be too low with its native promoter. We then generated a *GIL1*-GFP fusion construct under the control of the strong, constitutive RP27 promoter and transformed it into the protoplast of PH-1. GFP signals were observed in the resulting transformant R11 that was confirmed to contain the transforming *GIL1*-GFP construct by PCR. R11 had similar growth rate, colony morphology, and conidium morphology as the wild type strain PH-1 (Figure S3), suggesting that overexpression of *GIL1* had no obvious effects on growth and asexual reproduction of *F. graminearum*.

**Figure 6:**
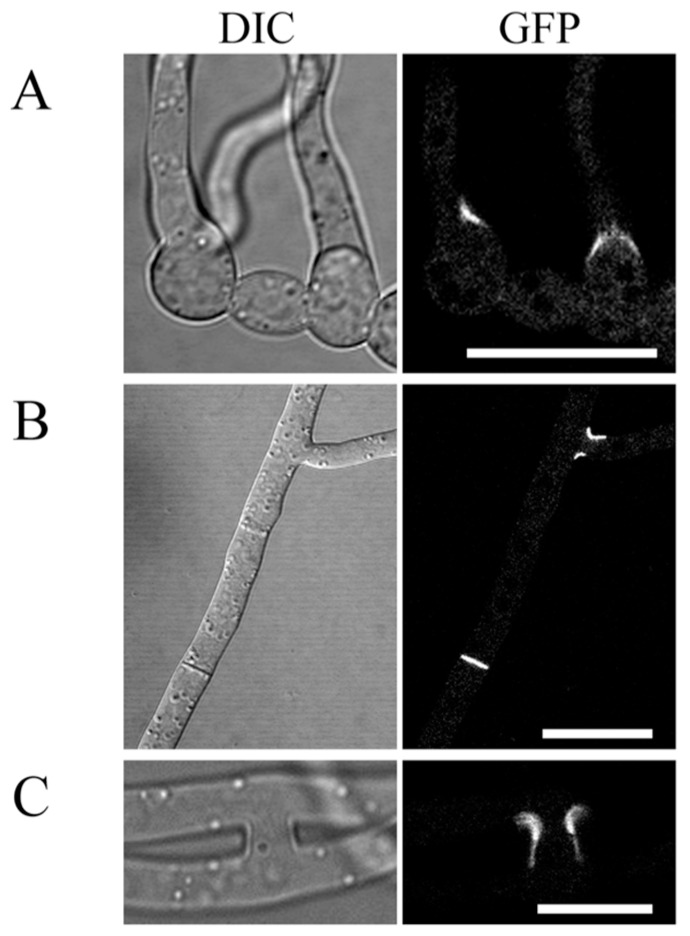
Subcellular localization of the GIL1-GFP fusion proteins in the P_RP27_-*GIL1*-GFP transformant R11 examined by differential interference contrast (DIC) and epifluorescence microscope (GFP). (**A**) In the germinated conidia (6 h), fluorescence signals (blight white) were observed at the base of the germ tubes; (**B**) fluorescence signals were observed at the septa and branching sites in the vegetative hyphae of R11; and (**C**) fluorescence signals localized at hyphal fusion site. For (**B**,**C**), the vegetative hyphae were from R11 colonies grown on MM (Minimal Medium) plate for 16 h. Bar = 10 μm.

During conidium germination, GFP signals were often observed at the base of the germ tubes (Figure 6A), suggesting that GIL1 may play a role in germ tube emergence and delineating germ tubes from the conidium compartments. In addition, GFP signals were occasionally observed at the septa (Figure 6B) in vegetative hyphae of R11. In repeated experiments, approximately 7% of the septa examined had GFP signals. Since not all the septa had GFP signals, the localization of GIL1 to the septum may be a transient or dynamic process. Interestingly, GIL1-GFP signals were often observed at the hyphal branching and hyphal fusion sites in the *GIL1*-GFP transformant R11 (Figure 6B,C), indicating possible roles of GIL1 in hyphal branching and fusion. The localization of GIL1-GFP fusion proteins to the septation and branching sites may be related to the defects of the Δ*gil1* mutant in hyphal growth and branching.

## 3. Discussion

*GIL1* encodes a protein with a typical serine/threonine protein kinase domain at the N-terminal region and a long C-terminal region without distinct motifs or domains. In the budding yeast, the C-terminal region of GIN4, HSL1, and KCC4 are responsible for their functional specificities. Therefore, it is likely that this region of GIL1 is involved in its interaction with other proteins, including the substrates of the GIL1 kinase. Interestingly, *GzSNF1* is the top hit of *GIN4*, *KCC4*, or *HSL1* in *F. graminearum*. Nevertheless, *GzSNF1* is an ortholog of yeast *SNF1* and it is essential for normal sexual and asexual development in addition to virulence and the utilization of alternative carbon sources [36].

In *F. graminearum*, the Δ*gil1* mutants were reduced in growth rate and formed whitish, compact colonies [13], which may be related to the hyper-branching defect observed at colony edges and germ tubes. Therefore *GIL1* likely plays a role in hyphal branching in *F. graminearum* and possibly in other filamentous ascomycetes. Hyphal branching is not well studied in *F. graminearum*. The Δ*Fgcla4* deletion mutant was increased in hyphal branching but it had much more severe defects in growth than the Δ*gil1* mutants. In *A. nidulans*, the Δ*aspA* and Δ*aspC* mutants are known to have the hyper-branching defects [37]. It is possible that the GIL1 kinase may be functionally related to these genes.

Interestingly, in comparison with the wild-type, conidium germination is delayed in the Δ*gil1* mutants. Therefore, *GIL1* may be involved in the establishment of polarized growth, which is similar to the function of GIN4, KCC4 and HSL1 in the budding yeast [22,24,26]. The Δ*kcc4* mutant has a multi-budded cell shape at stationary phase [26]. Nevertheless, hyphae of the Δ*gil1* mutants tended to be more curved than normal hyphae produced by the wild type under the same culture conditions. It is likely that *GIL1* also plays a role in the maintenance of polarized growth during hyphal elongation. Interestingly, the Δ*gil1* mutants had increased tolerance to cell membrane, cell wall and ROS stresses, suggesting that *GIL1* is involved in the cell wall integrality pathway and ROS response. In the budding yeast, the Δ*kcc4* mutant also has increased tolerance to 0.04% SDS [26]. Therefore, the *GIL1* in *F. graminearum* may have the functions of GIN4, KCC4 and HSL1 in the budding yeast.

In addition to hyphal growth, deletion of *GIL1* also impacted conidiogenesis in *F. graminearum*. The Δ*gil1* mutants were reduced in conidiation, which may be related to its defects in phialide formation. Instead of producing conidia efficiently on clusters of phialides, the Δ*gil1* mutants often formed conidia directly on short hyphal branches or at the hyphal tips (Figure 2A). In *F. graminearum*, the Δ*Fgcdc15*, Δ*Fg08631* and Δ*Fgrim15* mutants were also defective in phialide formation and had more severe defects in conidiation than the Δ*gil1* mutants [13]. Conidia formed by the Δ*gil1* mutants also had morphological defects, which often lacked foot cells and appeared to be straight.

Interestingly, Δ*gil1* conidia tend to be longer than the wild-type conidia in *F. graminearum*. The Δ*gin4* cells in the budding yeast were moderately elongated when cultured at 23 °C and pronounced longer when grown at 37 °C [22]. In *C. albicans*, the Δ*gin4* mutant also grew in chains of elongated cells, indicating a severe defect in cytokinesis [30]. Moreover, in comparison with the wild-type, some compartments of the vegetative hyphae in the Δ*gil1* mutant had longer septal distances and contained increased number of nuclei, suggesting the involvement of *GIL1* in regulation of cytokinesis in *F. graminearum*. Deletion of *GIN4* resulted in the production of significantly narrower hyphae in *A. gossypii* [31]. However, in *F. graminearum*, the Δ*gil1* mutants produced wider hyphae than that of the wild type. In *S. cerevisiae*, GIN4, KCC4, and HSL1 induce mitosis by releasing CDC28 from the inhibition of SWE1 [34]. Interestingly, the Δ*Fgswe1* mutant produced shorter conidia than the wild type [13], which is opposite to longer conidia produced by the Δ*gil1* mutants. Therefore, it is likely that the GIL1 kinase plays a similar role in counteracting the function of *FgSwe1* in *F. graminearum*.

The Δ*gil1* mutants were significantly reduced in virulence in infection assays with flowering wheat heads, corn stalks, and corn silks. Therefore, *GIL1* must play a critical role in pathogenesis in *F. graminearum*. Among all the phenotypes of the Δ*gil1* mutants characterized in this study, reduced growth rate may play a major role in contributing its defects in plant infection. However, the reduction of Δ*gil1* mutants in virulence was much more significantly than their reduction in growth rate. Other factors, such as changes in cell wall integrity may also affect virulence. *F. graminearum* is known to produce infection cushions, penetrating hyphae, and appressoria [38]. Considering its possible regulatory functions in cytokinesis, the GIL1 kinase may be important for the differentiation of plant infection structures, maybe involved in cell wall changes and other cellular differentiation events associated with these plant infection processes. In *C. albicans*, two *GIL1* homologs, *CaGIN4* and *CaHSL1*, have been identified but their roles in virulence were not characterized [30]. Interestingly, the *GIN4* and *NAP1* deletion mutants have similar phenotypes in *S. cerevisiae* [25]. In *C. albicans*, the *nap1* mutants are reduced in virulence [39], suggesting that the *Cagin4* mutants may be also defective in pathogenesis.

In *F. graminearum*, about 25% of the compartments in vegetative hyphae of the Δ*gil1* mutant showed longer septal distances than that of the wild-type, suggesting the possible role of *GIL1* in regulation of septum formation. In the budding yeast, the GIN4 kinase is involved in septin organization [22,25]. Its ortholog in *A. gossypii* is essential for septum formation because the Δ*gin4* mutant failed to produce septa in hyphae [31]. However, in *F. graminearum*, the number of septa in conidia was similar between the wild type and Δ*gil1* mutants. Therefore, the function of *GIL1* in septation remains to be clarified in different fungal cell types.

Interestingly, we observed that GIL1-GFP localized to the septum that separate the germ tube from conidium compartments, which is consistent with the defects of the Δ*gil1* mutant in conidium germination. In *S. cerevisiae*, GIN4 localizes to the bud neck [40] and deletion of *GIN4* results in abnormal septin deposition [22]. GIN4 is also required for the formation of the septin ring in *C. albicans* [30]. In *A. gossypii*, establishment of the inter-region (IR) septin rings is dependent on the GIN4 kinase [31]. In vegetative hyphae, GIL1-GFP signals were observed in some but not all the septa, indicating that localization of GIL1 to the septum is likely transient or dynamic. Moreover, GIL1-GFP signals were often localized at the branching and hyphal fusion sites, suggesting its possible roles in hypal branching and fusion. Because short specialized fusion branches are formed during the initiation of hyphal fusion of filament fungi [41], it is possible that localization of GIL-GFP at the hyphal fusion sites is related to formation of the fusion branches. Localization of GIL1 to the septum and branching sites is consistent with the defects of the Δ*gil1* mutants in septation and hyphal branching. It is likely that the GIL1 kinase is involved in the regulation of septum formation and initiation of hyphal branching in *F. graminearum* and possibly other fungi.

## 4. Materials and Methods

### 4.1. Strains and Culture Conditions

The wild-type strain PH-1, ectopic transformant, and Δ*gil1* mutant strains of *F. graminearum* were routinely cultured on potato dextrose agar (PDA), complete medium (CM), or 5× YEG plates at 25 °C as described [42,43]. Protoplast preparation and PEG (polyethylene glycerol)-mediated transformation of *F. graminearum* were performed as described [5,42]. To test response against various stresses, vegetative growth was assayed on CM plates with 0.7 M NaCl NaCl (Guangdong Guanghua Sci-Tech Co., Ltd., Shantou, China), 0.01% (*w*/*v*) SDS (MP Biomedicals, LLC, Solon, OH, USA), or 300 μg/mL Congo Red (SIGMA-ALDRICH Co., St. Louis, MO, USA) [13,21].

### 4.2. Identification of *Δ*gil1 Mutants

The Δ*gil1* mutants were generated with the split-marker approach in a previous study [13]. The 929-bp upstream flanking genomic sequence of *GIL1* and 592-bp genomic sequence of *GIL1* were amplified with primer pairs F1-R2 and F3-R4, respectively (Figure S2; Table S1). The resulting PCR products were connected to the hygromycin phosphotransferase (*hph*) cassette amplified with primers HYG/F-HY/R and YG/F-HYG/R (Table S1) by overlapping PCR and transformed into protoplasts of PH-1 as described [12]. For transformation selection, hygromycin B (Calbiochem, La Jolla, CA, USA) was added to the final concentration of 250 µg/mL to the regeneration medium. Putative Δ*gil1* mutants identified by PCR were confirmed by Southern blot hybridization analysis with *Kpn*I-digested genomic DNA. Surprisingly, in the wild type PH-1 and ectopic transformant E5, the bands detected with a *GIL1* fragment amplified with primers F5 and R6 (probe 1) are much larger than the expected 6.5 kb (Figure S2B; Table S1), which may due to failed or partial digestion. Instead of being the same size, the *GIL1* band of E5 was smaller than that of PH-1 (Figure S2B), indicating that the *GIL1* locus in E5 was partially modified by the *hph* integration event though the *GIL1* gene remained in its genome. The same probe had no hybridization signal in transformants T10 and T14 (Figure S2B). When probed with a fragment of the *hph* gene amplified with primers H850 and H852 (Table S1), PH-1 had no hybridization signals, and E5 and transformants T10 and T14 had a 3.0-kb band (Figure S2B). However, another *hph* band of 10.0 kb was also detected in T10 (Figure S2B), suggesting that multiple copies of the *hph* gene were present in T10. For PCR analysis of the *GIL1* and *hph* genes with genomic DNA of the *gil1* mutants (T10 and T14), E5, and wild-type (PH-1), a 1.07 kb band of the *GIL1* gene was amplified for E5 and PH-1, and a 0.75 kb band for the *hph* gene for T10, T14 and E5 strains (Figure S2C). In addition to the 0.75 kb *hph* band, there are aspecific *hph* amplicons present for T10 (Figure S2C), which may be caused by multiple close integrations of the *hph* gene. In total, both southern blotting and PCR analysis demonstrated that besides deletion of *GIL1*, at least one *hph* integration event is present in T10. Since the phenotypes of T10 are identical to that of T14, it is the deletion not the insertion that caused the phenotype of T10.

### 4.3. Assays for Growth and Conidiation Defects

Growth rate and colony morphology on PDA, CM and 5× YEG plates were measured after grown at 25 °C for three days. Conidiation and conidium morphology with five-day-old CMC cultures were examined as described [42,44]. Freshly harvested conidia were germinated in liquid YEPD medium for 3, 6, and 9 h, respectively, and examined for defects in conidium germination and germ tube growth.

### 4.4. Plant Infection Assays

For infection assays with wheat heads and corn stalks, conidia harvested from five-day-old CMC cultures were re-suspended to 10^5^ spores/mL in sterile distilled water, and 10 µL of conidium suspensions were used to inoculated each flowering wheat head of cultivar Norm as described [45]. To maintain moisture, inoculated wheat heads were capped with a plastic bag for 48 h. Disease symptoms were examined 14 dpi. Eight-week-old corn stalks of cv. Pioneer 2375 were inoculated with conidium suspensions by toothpicks as described [44,46]. Symptom development was assayed at 14 dpi by splitting the corn stalks along the inoculation sites. Infection assays with corn silks of cultivar Pioneer 2375 were conducted with culture plugs as described [17].

### 4.5. Generation of the GIL1-GFP Fusion Constructs

To generate the P_GIL1_-*GIL1*-GFP fusion construct, the *GIL1* gene with its 1.5-Kb upstream promoter sequence were amplified with primer GIL1/F and GIL1/R (Table S1) and cloned into pFL2 [47] by the yeast in vivo homologous recombination approach [48]. The P_RP27_-*GIL1*-GFP construct was generated with a similar approach by cloning the PCR product amplified with primers GIL1F/RP27 and GIL1R/RP27 (Table S1) into pFL2. The resulting *GIL1*-GFP construct was transformed into protoplasts of the wild type PH-1. Geneticin (MP Biochemicals, Santa Ana, CA, USA)-resistant transformants harboring the transforming GFP fusion constructs were identified by PCR and examined for GFP signals using a confocal microscopy with Nikon Tie system (Nikon, Japan).

## 5. Conclusions

Our data showed that the GIL1 kinase is important for different developmental stages of *F. graminearum*. It plays roles in hyphal growth, septation, conidiation, stress responses, and virulence. The hyphal branching and delayed conidium germination of the *GIL1* deletion mutants and localization of the GIL1-GFP fusion proteins suggest that GIL1 is involved in establishment of polarized growth, probably through affecting microtubule organization.

## Figures and Tables

**Table 1:** Phenotypes of the Δ*gil1* mutants in growth, conidiation, and conidia size.

Strain	Growth Rate (cm/day) ^a^			Conidiation (10^6^ conidia/mL)	Conidium Size (µm)	
	PDA	CM	5× YEG		Length	Width
PH-1	1.3 ± 0.0 ^A^	1.0 ± 0.0 ^A^	1.0 ± 0.0 ^A^	1.2 ± 0.1^A^	41.6 ± 0.7 ^B^	5.4 ± 0.2 ^A^
T10	0.8 ± 0.0 ^B^	0.8 ± 0.0 ^B^	0.7 ± 0.0 ^B^	0.7 ± 0.1^B^	49.2 ± 2.8 ^A^	5.4 ± 0.2 ^A^
T14	0.8 ± 0.0 ^B^	0.8 ± 0.0 ^B^	0.7 ± 0.0 ^B^	0.8 ± 0.1^B^	47.5 ± 2.0 ^A^	5.3 ± 0.4 ^A^

**^a^** Growth rate and conidiation were measured after incubation for three and five days, respectively. Mean and standard deviation were calculated from three independent replicates. Data were analyzed with Duncan’s multiple range test. The same capitalized letter indicated that there was no significant difference. Different capitalized letters were used to show statistically significant difference (*p* < 0.05). PDA, potato dextrose agar; CM, complete medium; YEG, Yeast Extract Glucose.

## Supplementary Materials

Supplementary materials can be found at www.mdpi.com/1422-0067/18/2/424/s1.
