# Supplementary material for: A Gin4-Like Protein Kinase GIL1 Involvement in Hyphal Growth, Asexual Development, and Pathogenesis in Fusarium graminearum
Source: Int J Mol Sci. 2017 Feb 16;18(2):424. doi: 10.3390/ijms18020424 (PMC5343958; doi:10.3390/ijms18020424)
Supplement: Supplementary file 1 [file ijms-18-00424-s001.pdf]

Supplementary Materials: A Gin4-Like Protein Kinase GIL1 Involvement in Hyphal Growth, Asexual Development, and Pathogenesis in *Fusarium graminearum*

Dan Yu, Shijie Zhang, Xiaoping Li, Jin-Rong Xu, Zachary Schultzhaus and Qiaojun Jin

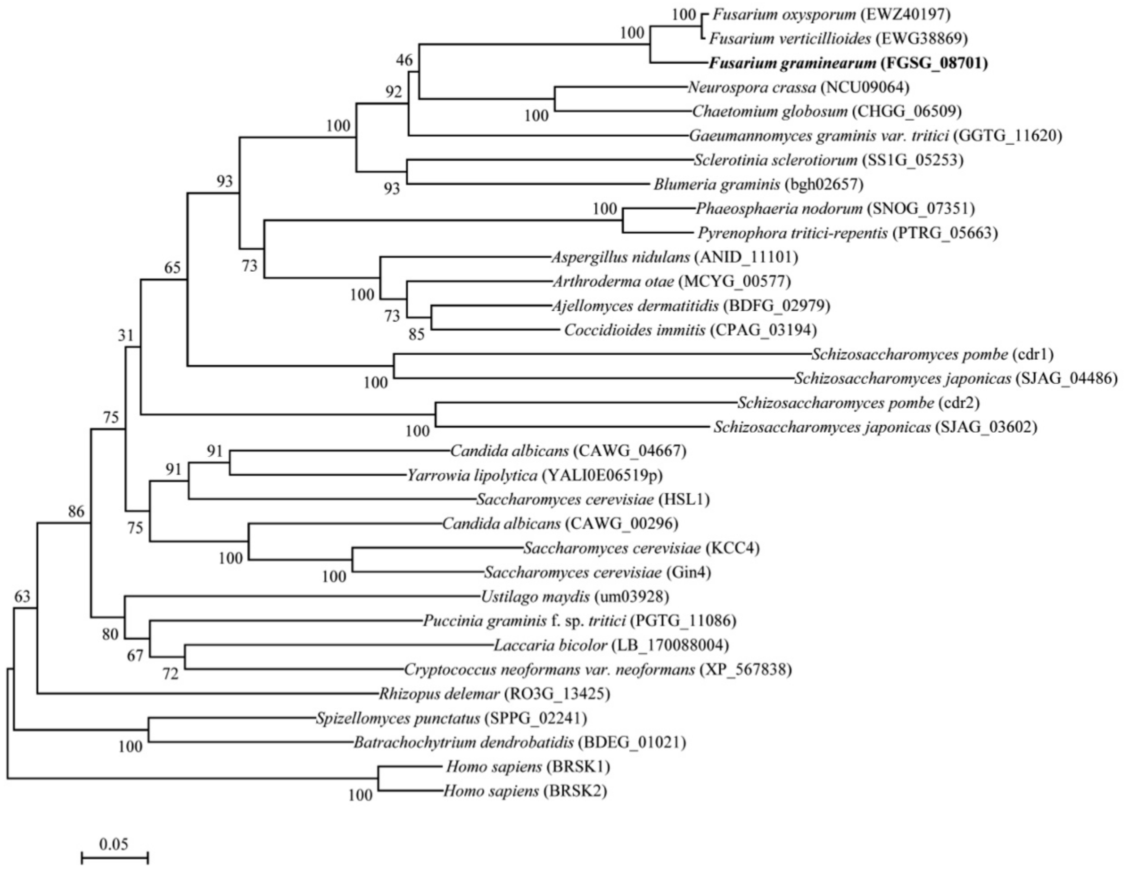

Figure S1. Phylogenetic analyses of *Fusarium graminearum* GIL1 and its homologs from other fungi. The amino acid 378 sequences of GIL1 (FGSG_08701) (Bold), and its homologs from the indicated fungal species were used to construct the phylogenetic tree based on Neighbor Joint analysis. Confidence of groupings was estimated by using 1000 bootstrap replicates. Numbers next to the branching point indicate the percentage of replicates supporting each branch.

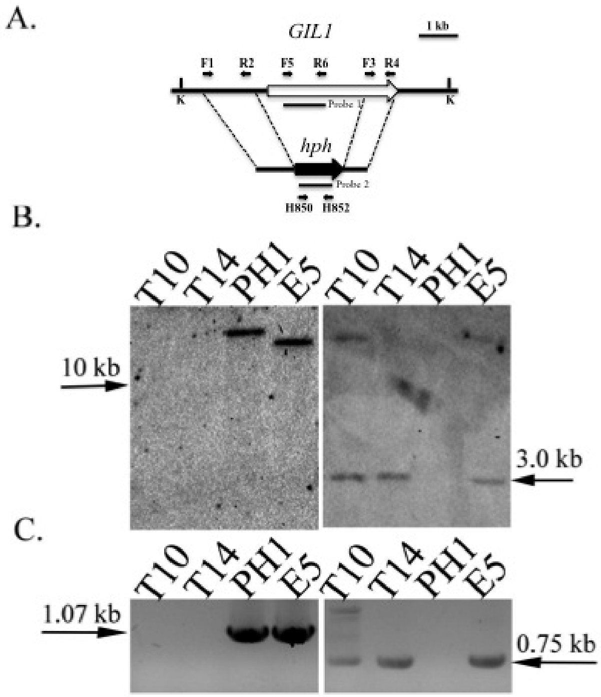

Figure S2. The *GIL1* gene replacement construct and Southern blot analysis. (**A**) Schematic draw of the *GIL1* gene, hygromycin-phosphotransferase (*hph*) cassette, the positions/directions of PCR primers, and the restriction enzyme sites.The *GIL1* and *hph* genes are marked with empty and black arrows, respectively. F1, R2, F3 and R4 are primers used to amplify the flanking homologous sequences. K, *Kpn* I; (**B**) Southern blots of *Kpn*I-digested genomic DNA of wild-type PH-1, Δ*gil1* mutants (strains T14 and T10), and an ectopic transformant (E5) were hybridized with probe 1 (left) amplified with primers F5 and R6, and probe 2 (right) amplified with H852 and H850; (**C**) PCR analysis ofthe *GIL1* and *hph* genes with genomic DNA of the Δ*gil1* mutants (T10 and T14), E5, and wild type (PH-1). The expected PCR products amplified by primer pairs F5/F6 (left panel) and H850/H852 (right panel) were labelled on the side.

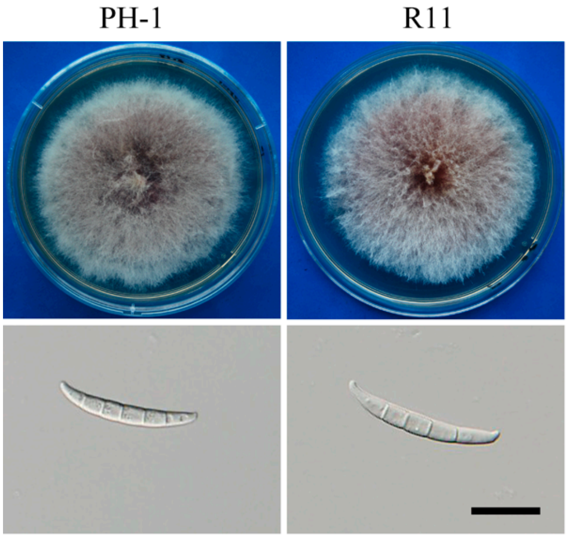

Figure S3. The phenotypes of PH-1 and the P_{RP27}-GIL1-GFP transformant R11. Colony morphology were assayed with PDA cultures grown at 25 °C for three days. Conidium morphology was examined with 5 day CMC cultures and photographed with an Olympus BX-51 microscope. Bar = 20 μm.

Table S1. Primers used in this study.

Name	Sequence (5′–3′)
F1	CTCTTGTGAGTTGGGTTGGCTTC
R2	TTGACCTCCTCTAGCTCCAGCCAAGCCTTGTCAAAATTATGTTCGCGCGT
F3	GAATAGAGTAGATGCCGACCGCGGGTTGCAAGCGAAGCGACTCTAC
R4	TGCCATGCTCAATCACCGT
F5	GCCGACCTTTGGAGTATGGGTG
R6	GGTAGGTTGATCCTTGTTCCGTGAC
GIL1/F7	TCCCGTCTTGGTTGTCTCAGTC
GIL1/R8	ATACTCCATAGTCACAAAGCCCA
HYG/F	GGCTTGGCTGGAGCTAGTGGAGGTCAA
HY/R	GTATTGACCGATTCCTTGCGGTCCGAA
YG/F	GATGTAGGAGGGCGTGGATATGTCCT
HYG/R	AACCCGCGGTCGGCATCTACTCTATTC
H855R	GCTGATCTGACCAGTTGC
H856F	GTCGATGCGACGCAATCGT
H852	AACTCACCGCGACGTCTGTC
H850	TTGTCCGTCAGGACATTGTT
GIL1/F	CGACTCACTATAGGGCGAATTGGGTACACAAATTGGTATCTAAACCAACTGTATGCTCC
GIL1/R	CACCACCCCGGTGAACAGCTCCTCGCCCTTGCTCACAGAGTTGAGCGTCTTGATCATC
GIL1 F/RP27	CAGATCTTGGCTTTCGTAGGAACCCAATCTTCAATGGCGGATATTATGATTCCG
GIL1 R/RP27	CACCACCCCGGTGAACAGCTCCTCGCCCTTGCTCACAGAGTTGAGCGTCTTGATCATC
